# Sensitivity of Neuroimaging Indicators in Monitoring the Effects of Interferon Gamma Treatment in Friedreich’s Ataxia

**DOI:** 10.3389/fnins.2020.00872

**Published:** 2020-10-09

**Authors:** Marinela Vavla, Filippo Arrigoni, Nicola Toschi, Denis Peruzzo, Maria Grazia D’Angelo, Sandra Gandossini, Annamaria Russo, Eleonora Diella, Stefania Tirelli, Roberto Salati, Alessandra Rufini, Ivano Condo, Roberto Testi, Andrea Martinuzzi

**Affiliations:** ^1^Istituto di Ricovero e Cura a Carattere Scientifico (IRCCS) E. Medea Scientific Institute, Bosisio Parini, Italy; ^2^Department of Women’s and Children’s Health, University of Padua, Padua, Italy; ^3^Department of Biomedicine and Prevention, University of Rome “Tor Vergata, ” Rome, Italy; ^4^Athinoula A. Martinos Center for Biomedical Imaging, Harvard Medical School, Boston, MA, United States; ^5^Fratagene Therapeutics, Rome, Italy

**Keywords:** neuroimaging, Friedreich’s ataxia, trial, fMRI, task fMRI, resting-state fMRI

## Abstract

The identification of efficient markers of disease progression and response to possibly effective treatments is a key priority for slowly progressive, rare and neurodegenerative diseases, such as Friedreich’s ataxia. Various imaging modalities have documented specific abnormalities in Friedreich’s ataxia that could be tracked to provide useful indicators of efficacy in clinical trials. Advanced MRI imaging (diffusion tensor imaging, DTI; functional MRI, fMRI; and resting-state fMRI, rs-fMRI) and retinal imaging (optical coherence tomography, OCT) were tested longitudinally in a small group of Friedreich’s ataxia patients participating in an open-label clinical trial testing the safety and the efficacy of 6-month treatment with interferon gamma. While the DTI indices documented the slow progression of fractional anisotropy loss, fMRI and rs-fMRI were significantly modified during and after treatment. The fMRI changes significantly correlated with the Scale for the Assessment and Rating of Ataxia, which is used to monitor clinical response. OCT documented the known thickness reduction of the retinal nerve fiber layer thickness, but there was no change over time. This pilot study provides indications for the potential utility of fMRI and rs-fMRI as ancillary measures in clinical trials for Friedreich’s ataxia.

## Introduction

Friedreich’s ataxia (FRDA) is the most frequent among the autosomal recessively inherited ataxias. It is caused by a GAA triplet expansion in the first intron of the *FXN* gene, leading to a deficit of frataxin, a mitochondrial protein involved in the Fe-S cluster assembly and in intracellular iron management (Campuzano et al., 1996). It is characterized by sensory deficits and impairment in limb coordination, leading to the progressive loss of ambulation and difficulty of speech, swallowing, and hand dexterity. In addition, other systems and organs can be involved, leading to progressive cardiomyopathy, diabetes, and impairment of visual and hearing functions (Koeppen and Mazurkiewicz, 2013). In spite of extensive research and numerous clinical trials, there is still no cure for FRDA. Possible therapeutic strategies currently under development range from anti-oxidants, iron chelators, mitochondrial enhancers, epigenetic modifiers, and neuroprotective cytokines to frataxin-specific approaches, such as frataxin RNA and protein stabilizers, frataxin protein replacement, and gene therapy approaches (Clay et al., 2019).

Interferon gamma (IFNγ), a naturally occurring cytokine which was shown to be able to boost frataxin expression both *in vitro* and in rodent models (Tomassini et al., 2012), has been tested in several clinical trials. This drug shows a good safety profile and variable levels of efficacy, depending on the different biochemical and clinical measures used (Seyer et al., 2015; Marcotulli et al., 2016; Lynch et al., 2019; Vavla et al., 2020).

A major issue when measuring a drug’s efficacy in clinical trials for FRDA, given the individual variability and the slow progression of the disease, is the generally poor sensitivity of the clinical scales used to quantify minor changes (Patel et al., 2016; Reetz et al., 2016). The use of objective instrumental measures and the inclusion of pre- and post- treatment observation time points, to clearly define natural disease progression, may be valuable to better monitor the effects of novel therapeutic strategies and support a more efficient clinical trial design. In this regard, MRI has been used as a useful marker to monitor disease progression and response to experimental treatments in FRDA. Longitudinal studies reported no volume changes over time but persistent and progressive white matter (WM) changes in the superior cerebellar peduncles (Mascalchi et al., 2016; Rezende et al., 2016). Two FRDA studies have used diffusion tensor imaging (DTI) in order to investigate the efficacy of erythropoietin in short-treatment trials by presenting bilateral widespread supratentorial fractional anisotropy (FA) increase and infratentorial axial diffusivity (AD) changes (Egger et al., 2014) and also gray matter volume increase in the pulvinar (Santner et al., 2014).

Finally, measurements of the retina, which is considered an extension of the central nervous system and reported to be reduced in thickness in FRDA, could represent a surrogate outcome of neuronal integrity. Optical coherence tomography (OCT) studies showed thinning of the retinal nerve fiber layer (RNFL), which correlates with disease severity and the patient’s reported outcome measures (Fortuna et al., 2009; Noval et al., 2012; Dağ et al., 2014; Thomas-Black et al., 2019).

We performed an open-label, one-step dose escalation phase II clinical trial to assess the safety and the efficacy of IFNγ in FRDA patients. The results were partly published and included a series of objective outcome measures intended to detect changes in multiple affected systems in FRDA (Vavla et al., 2020). The clinical and the cardiac indicators testified for a halt in disease progression and in cardiac wall thickness and pointed out a rebound effect after the termination of treatment. In the present manuscript, we report additional results of the clinical trial with regard to the neuroimaging outcome investigation. We assessed whether IFNγ treatment induced quantitative modifications of the cerebral structure and function as measured by MRI in our cohort of FRDA patients. We believe that our findings have general implications for clinical trial design in FRDA.

## Materials and Methods

### General Information

FRDA patients participating in an open-label, one-step dose escalation phase II clinical trial investigating the safety and the efficacy of IFNγ treatment were assessed before and after therapy with MRI and OCT measures as reported in the following paragraphs. The FRDA patients underwent an escalating IFNγ dose (from 100 μg for 2 weeks to 200 μg for the following 22 weeks) for an overall treatment duration of 24 weeks. The patients included had a genetic diagnosis of FRDA, whose age ranged 10–40 years. The exclusion criteria included unstable medical condition (heart, respiratory, liver, or kidney failure), myelosuppression, blood dyscrasia, hypersensitivity to latex and/or to IFNγ, exposure to erythropoietin within 3 years prior to the study recruitment, use of other medications (e.g., deferiprone, idebenone, vitamin B, etc.), pregnancy, and breastfeeding. Efficacy (clinical and instrumental assessments) and safety measures were performed at baseline (T0), 2 weeks (T2w), 3 months (T3), at the end of the treatment at 6 months (T6), and at the end of the 6-month follow-up period (T12). Additionally, the clinical and the neuroimaging assessments were administered ∼6 months prior to study initiation (T-6). Detailed information about the clinical study design, inclusion criteria, IFNγ administration, and safety measures have been reported elsewhere (Vavla et al., 2020). The study was approved by the IRB (no. 04215-CE) and registered at the ClinicalTrials.gov website (NCT03888664). All adult participants and legal guardians for underaged patients or parents provided written informed consent at the beginning of the study.

### Neuroimaging Protocol

Brain MR evaluations were performed four times for each patient (four time points): over 6 months prior to the treatment period (T-6), at the beginning (T0) and at the end (T6) of treatment, and 6 months after the end of treatment (T12).

All examinations were performed on the same 3T MRI scanner (Philips Achieva Scanner; Philips Medical System, Netherlands) equipped with a digital 32-channel head coil and included both structural (DTI) and functional as task-based fMRI and resting-state fMRI (RS-fMRI) measurements.

The following sequences were acquired:

– a 3D T1-weighted (T1w) MPRAGE high-resolution sequence (TE = 4 ms, TR = 8 ms, flip angle 8°, SENSE factor 2, voxel size 1 × 1 × 1 mm^3^, matrix size 288 × 288 × 170).
– a multi-shell diffusion gradient echo-planar (EPI) sequence (15 directions at *b* = 300 s/mm^2^, 53 directions at *b* = 1,100 s/mm^2^, eight volumes at *b* = 0 s/mm_2_, TE = 81 ms, TR = 9,324 ms, SENSE factor 2, SPIR fat suppression, voxel size 2.2 × 2.2 × 2.2 mm^3^, matrix size 112 × 112 × 80).
– a T2-weighted (T2w) structural volume with a fat-suppressed TSE sequence to correct DTI data for susceptibility-induced EPI distortion artifacts (TE = 100 ms, TR = 3,000 ÷ 5, 000 ms, flip angle 90°, SENSE factor 2, voxel size 1.5 × 1.5 × 1.5 mm^3^, matrix size 160 × 160 × 110).
– a conventional task-activation fMRI acquired with a single-shot EPI sequence [field of view (FOV) = 240 × 240 mm^2^, 40 slices interleaved without gap, voxel size 2.5 × 2.5 × 3.5 mm^3^, TE = 20 ms, TR = 2,000 ms, flip angle 85°, 178 volumes].
– a rs-fMRI sequence acquired with a single-shot EPI sequence (FOV = 240 × 240 mm^2^, 40 slices interleaved without gap, voxel size 2.5 × 2.5 × 3.5 mm^3^, TE = 20 ms, TR = 2,000 ms, flip angle 85°, 233 volumes).

### Diffusion-Weighted Imaging Processing

Diffusion-weighted imaging data processing was performed by using the TORTOISE software (Irfanoglu et al., 2017). The pre-processing pipeline included a motion-correction step, a correction of image distortions using the non-distorted T2w volume as a reference, realignment to the anterior/posterior commissure plane, and an up-sampling to a final voxel resolution of 1.5 × 1.5 × 1.5 mm. Data were visually inspected to detect residual artifacts and/or wrong preprocessing results. Corrupted volumes were discarded from the subsequent analyses. The DTI tensor was computed by fitting the mono-exponential model using a non-linear estimator on the corrected data (Basser and Pierpaoli, 1996; Koay et al., 2006), and the fractional anisotropy and the Mean Diffusivity (MD) maps were derived for each subject. A DTI study template was built from all subject tensors with the DTI-ToolKit software package (Zhang et al., 2007), which uses a spatial registration algorithm based on the diffusion tensor similarity to achieve a better alignment of WM structures. The transformation between each subject tensor and the template was computed and used to back-project the JHU atlas regions of interest (ROIs) from the template to the subject space. Mean FA and MD values were computed in selected regions (left and right corticospinal tracts and inferior, middle, and superior cerebellar peduncles) for statistical analyses.

### Task-Based fMRI Processing

**FIGURE 1:**
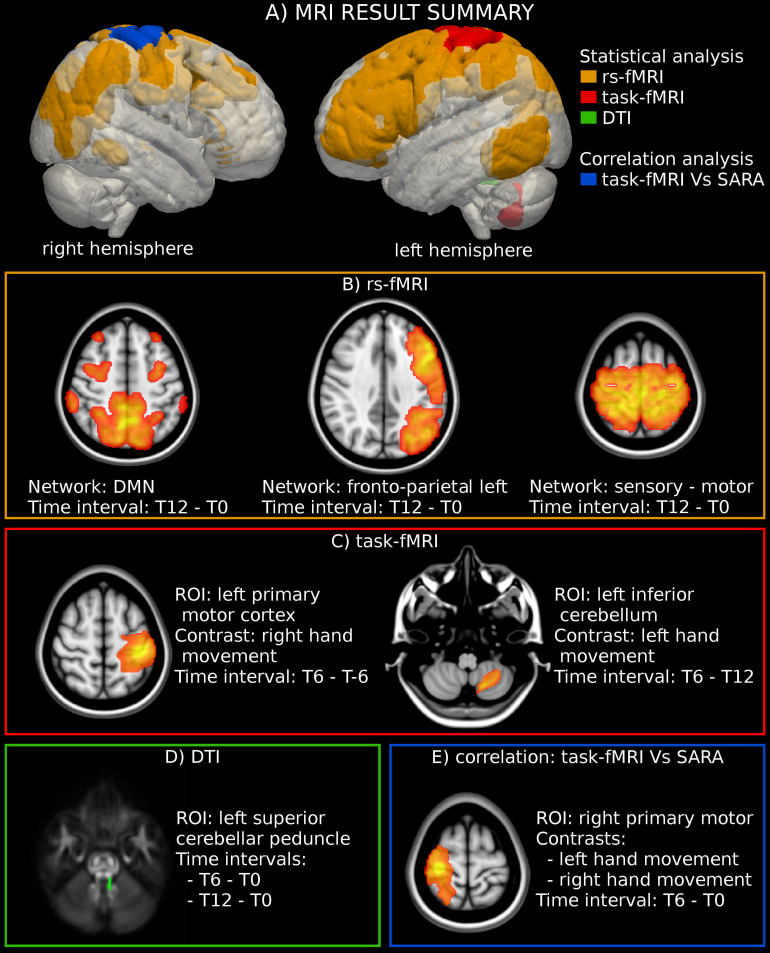
MRI analysis. **(A)** Location of the significant region of interest (ROIs) detected through the statistical and correlation analyses of MRI-derived measures. **(B)** Statistical analysis highlighted an activation increase in three rs-fMRI networks. The figure reports the result of the group-level statistical analysis used to define the networks’ ROIs (T-map superimposed to the MNI template). **(C)** The analysis of motor-task fMRI showed an increased activation in the left primary motor cortex during the movement of the right hand. The figure reports the statistical analysis used to define the ROI (T-map superimposed to the MNI template). **(D)** A significant reduction of fractional anisotropy (FA) was found in the left superior cerebellar peduncle. The figure reports the location of the left superior cerebellar peduncle superimposed to the FA template. **(E)** A significant negative correlation was found between the modification of SARA scores and the functional activity in the right motor cortex for both the dominant (right) and the non-dominant (left) hands. The figure reports the statistical analyses used to define the ROIs (T-map superimposed to the MNI template).

The task fMRI protocol was a standard block-design finger tapping task, involving both hands, as previously described (Vavla et al., 2018). The fMRI data for the motor-task were preprocessed by combining multiple tools (FSL, ANTs, and SPM). In particular, EPI volumes were realigned to the series mean image with a rigid body transformation using FSL’s MCflirt tool to correct for the subjects’ movements. A Gaussian filter with a full-width half-maximum (FWHM) equal to 6 mm was applied to the functional data to increase the signal-to-noise ratio (SNR). Additionally, a high-pass temporal filter with cutoff frequency of 128 s was used to correct for signal drifts. The general linear model approach was adopted to perform the single-subject analysis: the design matrix included one regressor for each state (rest, right hand movement, and left hand movement), six regressors for the rigid registration parameters of the motion correction process, and one regressor for each outlier volume. The ARtifact Detection Tool (ART)^1^ was employed to detect outliers: a volume was labeled as an outlier if it corresponded to a motion displacement greater than 3 mm or to a spurious intensity value (spikes). After the model estimation, we created a map for each effect that we wanted to test (i.e., contrast maps). In particular, we computed the contrast maps between each hand movement and the rest of the conditions for the statistical analysis (named “dominant hand” and “non-dominant hand”). Several transformations were computed to move the contrast maps into a template space and perform a group-level analysis. In particular, we computed a non-linear registration between the functional mean image and the undistorted T2w of the subject, a rigid transformation between the subject T2w and the subject T1w, and a non-linear transformation between the subject T1w and the MNI152 template (Nordio et al., 2016). Finally, all transformations were combined together to get one single transformation going to the subject functional space to the MNI152 space. All contrast maps were moved to the MNI152 space using the combined transformation defined above and were subsequently used as input for the group-level analyses. Following the same rationale of the statistical analysis performed on DTI data, a ROI-based analysis onto contrast maps was also performed. The ROIs were delineated on the basis of the activation clusters generated by hand movement comparisons (contrast “dominant hand > non-dominant hand” and “non-dominant hand > dominant hand”) using a “one-sample *t*-test” considering all subjects and all the time points together. Significance was set to *p* < 0.05, corrected for multiple comparisons with the FWE method. We selected four ROIs related to the “dominant > non-dominant” contrast and four ROIs related to the “non-dominant > dominant” contrast (Figure 1A). Significance was set to *p* < 0.05, corrected for multiple comparisons with the FWE method. For each ROI, the mean intensity value was computed for the “dominant hand” and the “non-dominant” contrasts for statistical analyses.

### rs-fMRI Processing

During the resting-state acquisition (rs-fMRI), the subjects were instructed to refrain as much as possible from any active cognitive activity and to keep their eyes closed. The rs-fMRI sequence was performed at the end of the MR protocol. Following the same preprocessing steps of the task fMRI data described above, the functional volumes underwent a motion correction procedure, and the transformation between the subject functional space and the MNI152 template space was computed as described for the task fMRI protocol. Subsequently, the mean signals in the cerebrospinal fluid, WM, and motion parameter were regressed out. A band-pass filter (0.008–0.01 Hz) was applied to remove physiological and non-BOLD-related effects. Finally, functional sequences were smoothed with a Gaussian spatial filter (FWHM, 4 mm) to increase the SNR and to deal with the residual anatomical differences between subjects. Independent component analysis (ICA) was conducted using FSL MELODIC software by multi-session temporal concatenation. Automatic procedure estimation was used to select 24 ICA group components. The selection of “good” and “bad” components was performed by visual inspection of the spatial pattern of ICA maps and the power spectrum of the time courses. This procedure led us to label seven components as related to resting-state networks (RSNs). The selected components refer to the default mode network (DMN), the hippocampus network, the right and the left fronto-parietal networks, the bilateral fronto-temporal network, the visual network, and the motor network. Using the dual-regression method, we used this set of group-average spatial maps to generate a corresponding set of subject-specific spatial maps and time-series for each subject. We then obtained one spatial ICA map and one-time signal for each subject and each group-ICA component. Furthermore, a component-specific ROI was computed to restrict the group analysis to the voxels in that component. To do that, a one-sample *t*-test was run using all single subjects’ component maps with SPM12 (significance was set to *p*RSN < 0.05, corrected for multiple comparisons with the FWE method). Finally, for each RSN, the subject value in each time point was defined as the mean intensity values in the component-specific ROI and used for the subsequent statistical analyses.

### Optical Coherence Tomography

All eyes were examined with spectral domain OCT (Optovue, United States) at T0, T6, and T12. The program studied both the peripapillary area and the macula, following pupillary dilation. The RNFL thickness and the ganglion cell complex (GCC) thickness were evaluated in the peripapillary area by a circular scan centered on the optic disk (3.45 mm diameter, “disk circle” option).

### Statistical Analysis

All variables and biomarkers were analyzed using multivariate linear mixed models which modeled time points as a repeated within-subject factor and a Toeplitz unstructured estimate of the covariance matrix. The mixed models have the advantage of being able to account for heterogeneous distances between time points, missing data as well as unequal variances and covariances. In order to account for possible confounds due to inter-patient variability, all models included sex, age at onset, disease duration, years of education, and number of GAA1 repeats within the smaller *FXN* allele as covariates of no interest. When a statistically significant (*p* < 0.05) overall effect of time was found, pairwise comparisons between time points were performed and corrected for multiple comparisons across pairs of time points using the Dunn–Šidák correction. The analysis was also repeated without the latter correction in order to identify possible trends. Marginal means for each variable and timepoint were also estimated from the models and evaluated at mean values at the covariates of no interest. Moreover, we evaluated the correlations (Pearson test) between the modifications in SARA scores between consecutive time points and the respective changes in the MRI-derived structural and functional measures.

## Results

### Clinical Results

Detailed clinical and demographic data have been published elsewhere (Vavla et al., 2020). Twelve patients with a mean age of 17.33 ± 4.54 years were recruited and 11 completed the study. During the IFNγ treatment, non-significant and slightly negative changes in the SARA score were measured with a moderate uptrend in the follow-up period, suggestive of efficacy. The cardiac parameters registered a reduction of the end-diastolic interventricular septal wall thickness and the Sokolow–Lyon index during the treatment period, with a rebound effect in the follow-up period. The frataxin quantitation in peripheral blood mononuclear cells did not provide significant changes.

### Magnetic Resonance Imaging Results

WM DTI analysis revealed a significant reduction of FA values in the left superior cerebellar peduncle at T12 (*p*-value = 0.019 corrected) vs. T0 in 11 patients. No significant changes could be documented along the intermediate time points nor corresponding to the exposure to IFNγ treatment (a trend of FA reduction with an uncorrected *p*-value = 0.015 was observed between T0 and T6) (Figure 1D, Table 1 and Supplementary Table S1). MD did not show any significant differences among timepoints.

**TABLE 1:** Diffusion tensor imaging data quantitating the mean fractional anisotropy (FA) in the left superior cerebellar peduncle (LSCP) at different time points in 11 patients.

**LSCP**	**T-6**	**T0**	**T6**	**T12**
Mean	0.431 ± 0.014	0.440 ± 0.010	0.429 ± 0.011	0.423 ± 0.099
FA (μ^2^/s)				

*A general decrease was observed over time and a significant difference was measured between T0 and T12 (p = 0.019, corrected). Other comparisons were not significant. Mean FA measured in other brain regions did not show significant changes over the different time points.*

**FIGURE 2:**
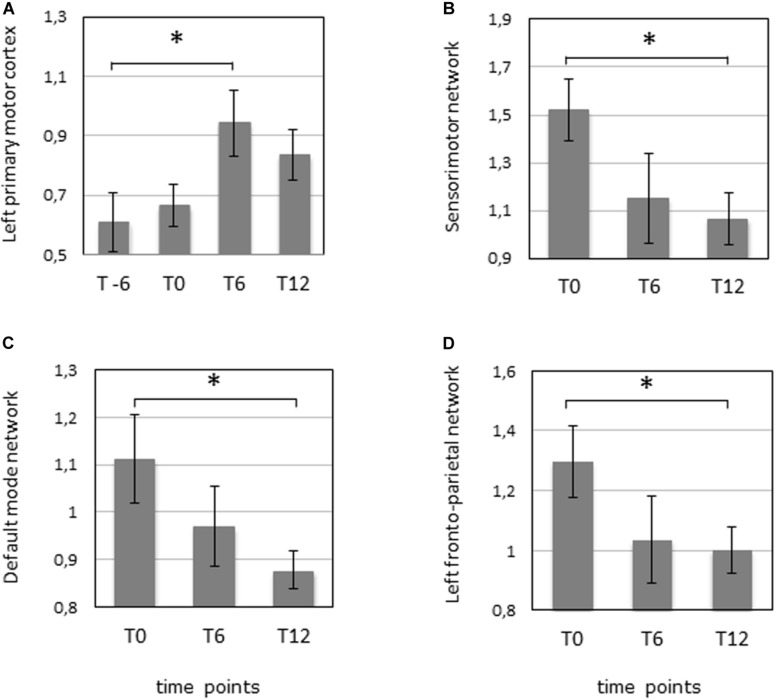
fMRI was performed 6 months before the start of the IFNγ treatment (T-6), before the start of the treatment (T0), after 6 months of treatment (T6), and 6 months after the termination of treatment (T12) in 10 patients. **(A)** Task fMRI measuring the mean activation in the left primary motor cortex during the dominant (right) hand movement. ^∗^*p* = 0.047 (corrected). **(B)** Resting-state fMRI measuring the mean activation of the sensorimotor network. ^∗^*p* = 0.027 (corrected). **(C)** Resting-state fMRI measuring the mean activation of the default mode network. ^∗^*p* = 0.031 (corrected). **(D)** Resting-state fMRI measuring the mean activation of the left fronto-parietal network. ^∗^*p* = 0.045 (corrected).

The analysis of motor-task fMRI (*n* = 10) showed an increased activation of the left primary motor cortex between T-6 and T6 during the movement of the dominant hand (*p*-value = 0.047 corrected). A similar trend was noted in the same area between T0 and T6 (*p*-value = 0.045 uncorrected) during movement of the non-dominant hand, as well as in the left inferior cerebellum between T6 and T12 (*p*-value = 0.03 uncorrected) for the non-dominant hand movement (Figures 1C, 2A, and Supplementary Table S1).

Three RSN networks such as sensorimotor network, DMN, and left fronto-parietal network showed a significantly modified activity between T0 and T12 with corrected *p*-values = 0.027, 0.031, and 0.045, respectively (*n* = 10). All the other comparisons were not significant (Figures 1B, 2B–D, and Supplementary Table S1).

**FIGURE 3:**
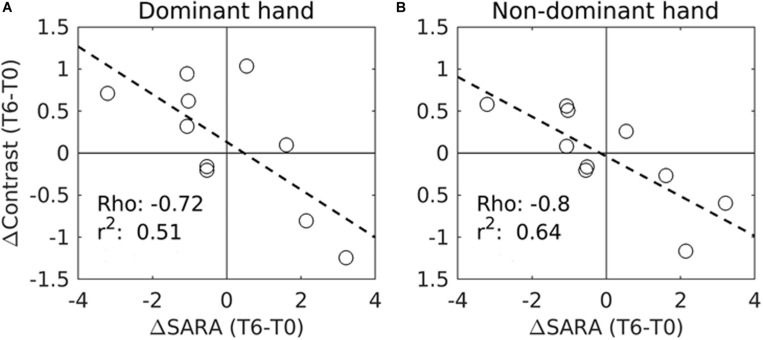
Correlation between the variations of SARA scores (ΔContrast) and the variations of the task fMRI activation measures (ΔContrast) in the right motor cortex during IFNγ treatment (from T0 to T6) in 10 patients. **(A)** fMRI measures were performed during dominant (right) hand movements. **(B)** fMRI was performed during non-dominant (left) hand movements. Significant correlations were found with the SARA scores for both the dominant hand movement (*p* = 0.02, corrected) and the non-dominant hand movement (*p* = 0.006, corrected). Circles represent individual patients. Rho and *r*^2^ values are also shown for each trend line (- - -). Due to the excessive motion artifacts and collaboration problems during acquisition, one patient was excluded from the motor-task fMRI analyses.

Importantly, a significant negative correlation was found between the modification of SARA scores and the functional activity in the right motor cortex for both the dominant and the non-dominant hands between T0 and T6 (Figures 1E, 3A,B).

### Optical Coherence Tomography

The RNFL and GCC thickness were measured by OCT before the start of the IFNγ treatment (T0), at the end of treatment (T6), and after the follow-up period (T12) in 11 patients. No significant changes were found when considering the average RNFL thickness or the RNFL thickness of the different quadrants considered separately (inferior, temporal, nasal, and superior quadrant) or the average GCC thickness for both eyes (Supplementary Table S2).

## Discussion

Here we describe the functional and the structural modifications occurring in the brain during and after an experimental treatment with IFNγ in a cohort of FRDA patients, with complementary results in support to the significant changes observed with disease-specific clinical measures (SARA and cardiac outcomes) (Vavla et al., 2020).

The structural WM results, measured by DTI, revealed a significant reduction of FA in the left superior cerebellar peduncle within a year, without distinction between the treatment and the out-of-treatment periods. Reduced FA values in the superior cerebellar peduncles have frequently been observed in FRDA and represent a structural hallmark of this disorder (Akhlaghi et al., 2011; Della Nave et al., 2011; Clemm von Hohenberg et al., 2013; Vavla et al., 2018). The progressive reduction of FA values that we observed is consistent with previous longitudinal studies. Without a placebo control group, we cannot make definitive considerations on this result, however, we can hypothesize that degenerative processes at the WM level continue to progress despite IFNγ treatment. The motor-task fMRI showed an increased activation of the left primary motor cortex during dominant hand movement across pre- and within-treatment periods. Trends of increasing activity (not reaching the significance level) were also detected in the left cerebellar cortex within the treatment period. The rs-fMRI showed a modified activation of three networks (DMN, sensorimotor, and left fronto-parietal) within 1 year from the beginning of IFNγ administration. These fMRI findings appear clearly associated to an increased activation of supratentorial and infratentorial areas involved in the motor task. The rs-fMRI analysis pointed toward a reorganization of different sensorimotor and executive networks during and after the treatment. It is interesting to note that such modifications seem to particularly involve only the dominant hand.

Differently from DTI modifications that imply structural and architectural changes at the cellular and the axonal levels, the fMRI differences may be the result of a reorganization of neuronal circuitry, which may be mediated by several factors like neurotransmitters, synaptic changes, or recruitment of new pathways. This may partially explain the changes in association with treatment in functional but not structural measurements. Moreover, a negative correlation between the enhanced motor cortex activation and the progression of the SARA score was detected within the treatment period, suggesting that the fMRI modifications that we observed during both hand movements corresponded to a more generalized clinical improvement. A correlation analysis suggests that the reorganization of neural circuits is underway during the period of drug administration, in parallel with an improvement of SARA scores. However, absolute differences in the measured activity of neural networks were only evident within a 1-year period, including the follow-up. The physiological implications of these findings require caution as the precise evolution of the brain activation pattern during disease progression has not yet been firmly established. The data that we collected point toward a possible effect of the IFNγ on the dynamics of neural activity, especially in motor areas and within a time-span that is longer than the actual treatment period. fMRI has been widely used to explore brain connectivity in many psychiatric and neurological disorders such as Alzheimer disease, Parkinson’s disease, and epilepsy. Few clinical studies have used fMRI to build a model useful to investigate treatment-related responses.

Therefore, it is important to understand the intrinsic network dynamics and investigate whether the disruption can be restored or modified after treatment (Smucny et al., 2014). Indeed Tregellas et al. (2011) have used DMN activity as a biomarker of treatment in schizophrenia, hypothesizing it as a predictor to treatment response. These studies add interesting hints to the future clinical trial design in FRDA.

We confirmed a general reduction in RNFL thickness in FRDA patients; nevertheless, no significant progression or change was observed during treatment or in a 1-year time-lapse. This could indicate that RNFL thickness would not appear to be an efficient marker for response to treatment in such a short period. So far, only one study has longitudinally investigated this measure in FRDA, with a surprising progression of RNFL thinning over a 6-month period (Rojas et al., 2020). These different results could be due to the fact that the cohort of Rojas et al. (2020) included older patients when compared to ours. Perhaps the RNFL impairment and the ocular involvement are age-related events that progress over time. Our group has recently tested the longitudinal changes in RNFL thickness in another neurodegenerative condition, hereditary spastic paraplegia, and found no changes over a 10-month period (Vavla et al., 2019). On the other hand, a 2-year longitudinal clinical trial in multiple sclerosis registered OCT changes and correlation to brain atrophy (Winges et al., 2019).

The main limitations of this work are the open-label design, the small sample size, and the relatively short time of observation. These limitations might have restrained our ability to document subtle changes. Although the different time-lapses were relatively short, we believe that this study offers information regarding the imaging modalities that are more likely to reflect changes in cerebral organization as a result of treatment in a neurodegenerative condition such as FRDA. We are far from claiming for fMRI indexes to be undoubtedly useful biomarkers/endpoints for FRDA treatments. We believe that the changes registered and the activation trends should be interpreted with some caution but still deserve attention as they documented significant modifications occurring in the brain and should be further validated in future studies.

In conclusion, this study explores the usefulness and the sensitivity of several imaging parameters to monitor disease progression during a treatment associated with significant clinical changes. These preliminary data encourage the use of MRI, in particular fMRI, as a non-invasive method to monitor treatment response in FRDA and to achieve a better understanding of the physiology behind the observed treatment-induced effects.

## Data Availability Statement

The datasets generated for this study are available on request to AM, andrea.martinuzzi@lanostrafamiglia.it.

## Ethics Statement

The study was approved by the IRB (No. 04215-CE) of Eugenio Medea IRCCS Research Center. Written informed consent to participate in this study was provided by the adult participant directly of by the participants’ legal guardian/next of kin.

## Author Contributions

AM, MV, FA, and RT: study concept and design. AM, FA, MV, and MD’A: conduct the research and study supervision. MD’A, SG, AnR, ED, and RS: patients’ assessment and clinical data gathering. FA, NT, DP, MV, AlR, IC, and RT: analysis and interpretation of data. FA, DP, ST, and NT: analysis of MRI data. FA: supervision of MRI analysis. MV, AM, and MD’A: enrolment of patients. MV, FA, NT, DP, MD’A, SG, AnR, ED, ST, RS, AlR, IC, RT, and AM took responsibility for manuscript writing and review.

## Conflict of Interest

AR and RT were employed by company Fratagene Therapeutics. The remaining authors declare that the research was conducted in the absence of any commercial or financial relationships that could be construed as a potential conflict of interest.

## Footnotes

1 http://web.mit.edu/swg/software.htm

## Supplementary Material

TABLE S1 Multivariate linear mixed model of MRI data (diffusion tensor imaging, task fMRI, rs-fMRI) run out with and without covariates of interest (sex, age at onset, disease duration, years of education, and GAA1 size). The results are presented as uncorrected and corrected. DMN, default model network; R, right; L, left; FA, fractional anisotropy; F, frontal; P, parietal; SCP, superior cerebellar peduncle; SM, sensosorimotor.

TABLE S2 Average retinal nerve fiber layer (RNFL) and ganglion cell complex thickness were measured by optical coherence tomography in the right eye (RE) and the left eye (LE). RNFL thickness is also reported for the temporal (Tem), superior (Sup), nasal (Nas), and inferior (Inf) quadrants of each eye in 11 patients. Changes among the reported mean ± SEM at different time points were not significant.
