# Supplementary material for: Sensitivity of Neuroimaging Indicators in Monitoring the Effects of Interferon Gamma Treatment in Friedreich’s Ataxia
Source: Front Neurosci. 2020 Oct 9;14:872. doi: 10.3389/fnins.2020.00872 (PMC7583645; doi:10.3389/fnins.2020.00872)
Supplement: TABLE S1 — Multivariate linear mixed model of MRI data (diffusion tensor imaging, task fMRI, rs-fMRI) run out with and without covariates of interest (sex, age at onset, disease duration, years of education, and GAA1 size). The results are presented as uncorrected and corrected. DMN, default model network; R, right; L, left; FA, fractional anisotropy; F, frontal; P, parietal; SCP, superior cerebellar peduncle; SM, sensosorimotor. [file Table_1.pdf]

Supplementary Table 1. Multivariate linear mixed model of MRI data (DTI, task-fMRI, rs-fMRI) run out with and without covariates of interest (sex, age at onset, disease duration, years of education, GAA1 size). Results are presented as uncorrected and corrected.

				With Covariates	**Uncorrected**		***No covariate***	**Uncorrected**	
**Variable**	**Timepoint measures**	**Marginal Mean**	**Std. Error**	***Overall test p-value***	**Pairwise comparison**	***Paiwise p-value***	***Overall test p-value***	**Pairwise comparison**	***Paiwise p-value***
**DMN_DMN**	.0	1,113	0,093	***0,017***	0 vs 12	***0,011***	***0,017***	0 vs 12	***0,011***
	6.0	0,969	0,085						
	12.0	0,877	0,040						
**Component_L_FP**	.0	1,295	0,120	***0,037***	0 vs 12	***0,015***	***0,037***	0 vs 12	***0,015***
	6.0	1,034	0,146						
	12.0	1,001	0,077						
**Component_SM**	.0	1,520	0,131	***0,013***	0 vs 12	***0,009***	***0,013***	0 vs 12	***0,009***
	6.0	1,151	0,188						
	12.0	1,065	0,111						
**Contrast_R-hand_L_SM_cortex**	-6.0	0,609	0,099	***0,012***	-6 vs 6	***0,008***	***0,012***	-6 vs 6	***0,008***
	.0	0,663	0,071		0 vs 6	***0,045***		0 vs 6	***0,045***
	6.0	0,943	0,111						
	12.0	0,835	0,085						
**FA_L_SCP**	-6.0	0,431	0,014	***0,027***	0 vs 6	***0,015***	***0,027***	0 vs 6	***0,015***
	.0	0,440	0,010		0 vs 12	***0,003***		0 vs 12	***0,003***
	6.0	0,429	0,011						
	12.0	0,423	0,011						
**Contrast_L-hand_L_Inferior_Cerebellum**	-6.0	0,287	0,091	***0,049***	6 vs 12	***0,030***	***0,049***	6 vs 12	***0,030***
	.0	0,414	0,087						
	6.0	0,439	0,064						
	12.0	0,247	0,045						

				With Covariates	Corrected		*No covariates*	Corrected	
	**Timepoint measures**	**Marginal Mean**	**Std. Error**	***Overall test p-value***	**Pairwise comparison**	***Paiwise p-value***	***Overall test p-value***	**Pairwise comparison**	***Paiwise p-value***
**DMN_DMN**	.0	1,113	0,093	***0,017***	0 vs 12	***0,031***	***0,017***	0 vs 12	***0,031***
	6.0	0,969	0,085						
	12.0	0,877	0,040						
**Component_L_FP**	.0	1,295	0,120	***0,037***	0 vs 12	***0,045***	***0,037***	0 vs 12	***0,045***
	6.0	1,034	0,146						
	12.0	1,001	0,077						
**Component_SM**	.0	1,520	0,131	***0,013***	0 vs 12	***0,027***	***0,013***	0 vs 12	***0,027***
	6.0	1,151	0,188						
	12.0	1,065	0,111						
**Contrast_R-hand_L_SM_cortex**	-6.0	0,609	0,099	***0,012***	-6 vs 6	***0,047***	***0,012***	-6 vs 6	***0,047***
	.0	0,663	0,071						
	6.0	0,943	0,111						
	12.0	0,835	0,085						
**FA_L_SCP**	-6.0	0,431	0,014	***0,027***	0 vs 12	***0,019***	***0,027***	0 vs 12	***0,019***
	.0	0,440	0,010						
	6.0	0,429	0,011						
	12.0	0,423	0,011						

Legend: DMN: Default Model network, R: right, L: left, FA: fractional anisotropy, F: frontal, P: parietal, SCP: superior cerebellar peduncle, SM: sensosorimotor.

Supplementary Table 2. Average Retinal Nerve Fiber Layer (RNFL) and Ganglion Cell Complex (GCC) thickness were measured by Optical Coherence Tomography in the right eye (RE) and left eye (LE). RNFL thickness is also reported for the temporal (Tem), superior (Sup), nasal (Nas) and inferior (Inf) quadrants of each eye in 11 patients. Changes among the reported Mean ± SEM at the different time points were not significant.

	**T0**	**T6**	**T12**
**RNFL RE Average (μm)**	80.0 ± 2.1	76.8 ± 3.0	75.8 ± 2.9
**RNFL LE Average (μm)**	84.7 ± 4.8	78.5 ± 3.2	77.3 ± 3.0
**RNFL RE Tem (μm)**	63.8 ± 5.7	67.6 ± 2.6	66.3 ± 2.9
**RNFL LE Tem (μm)**	67.2 ± 5.3	64.6 ± 3.4	62.4 ± 3.0
**RNFL RE Sup (μm)**	92.4 ± 5.4	91.4 ±3.5	88.9 ± 2.9
**RNFL LE Sup (μm)**	108.8 ± 9.8	91.6 ± 3.3	93.1 ± 3.4
**RNFL RE Nas (μm)**	67.6 ± 5.6	57.6 ± 3.2	56.3 ± 2.5
**RNFL LE Nas (μm)**	67.9 ± 11.7	60.2 ± 4.8	56.9 ± 2.7
**RNFL RE Inf (μm)**	95.6 ± 5.2	95.7 ± 3.7	95.0 ± 4.0
**RNFL LE Inf (μm)**	99.9 ± 8.3	101.7 ± 3.6	96.5 ± 3.4
**GCC RE Average (μm)**	88.5 ± 1.9	88.5 ± 1.3	90.9 ± 2.3
**GCC LE Average (μm)**	97.9 ± 8.8	88.7 ± 1.5	88.5 ± 1.7
